# Antibacterial and Photocatalytic Activities of *Leonotis ocymifolia* (*L. ocymifolia*)-Mediated ZnO Nanoparticles Annealed at Different Temperatures

**DOI:** 10.3390/nano14231929

**Published:** 2024-11-29

**Authors:** Dorcas Mutukwa, Raymond Tichaona Taziwa, Shepherd Masimba Tichapondwa, Lindiwe Khotseng

**Affiliations:** 1 Department of Chemistry, University of Western Cape, Robert Sobukwe Road, Private Bag X17, Bellville 7535, South Africa; lkhotseng@uwc.ac.za; 2 Department of Applied Sciences, Faculty of Natural Sciences, Walter Sisulu University, Old King William Town Road, Potsdam Site, East London 5200, South Africa; 3 Department of Chemical Engineering, Sustainable Environmental and Water Utilization Processes Division, University of Pretoria, Pretoria 0028, South Africa; shepherd.tichapondwa@up.ac.za

**Keywords:** green synthesis, plant-mediated, biomolecules, antimicrobial, antibacterial agents, photocatalysis, photodegradation, methylene blue, dyes, biosynthesis, photocatalyst

## Abstract

This research achieved the successful synthesis of zinc oxide (ZnO) NPs through an eco-friendly method, utilizing the leaf extract of *Leonotis ocymifolia* (L.O.). This innovative approach not only highlights the potential of green synthesis but also underscores the effectiveness of natural resources in nanoparticle production. The influence of annealing temperature on the properties and performance of the synthesized ZnO NPs was evaluated by varying the annealing temperatures as follows: unannealed (000), 350 °C (350), 550 °C (550), and 750 °C (750). The XRD analysis of L.O-mediated ZnO NPs confirmed the synthesis of highly crystalline wurtzite-structured ZnO NPs, with calculated average crystallite sizes that ranged between 13.8 and 20.4 nm. The UV–Vis spectra revealed a single strong absorption peak ranging from 354 to 375 nm, and the absorption peaks red-shifted with an increase in annealing temperature. The SEM micrographs showed that annealing temperature had an effect on the morphology, particle size, and distribution, with the average particle of 53.7–66.3 nm. The BET analysis revealed that the surface area of the prepared ZnO NPs was between 31.6 and 13.2 m^2^/g. In addition to its significant impact on the characteristics of the L.O-mediated, annealing temperature notably boosts the L.O-mediated capacity to photodegrade Methylene blue (MB) dye. Moreover, it exhibited significant antibacterial efficacy against *Escherichia coli* (*E. coli*) and *Staphylococcus aureus* (*S. aureus*). The photodegradation studies under UV irradiation and in 180 min revealed 750 (71.1%) had the highest degradation efficiency compared to 000, 350, and 550. The antibacterial tests showed that 000 had greater antibacterial efficacy than 350, 550, and 750. The results from this work suggest that annealing temperature had a significant effect on the structural, morphological, and optical properties and performance of L.O-mediated ZnO NPs.

## 1. Introduction

Dyes are essential components of industrial processes, and the rapid global urbanization and industrialization over the years have led to the increased presence of dyes in industrial wastewater. In Africa, industries such as paint, textile, tanner, food and artisan, and dye manufacturing have been identified as the main contributors to dye wastewater that deteriorates the water quality [1]. Dye effluents increase the chemical and biological oxygen demand (COD) and (BOD) of water systems, leading to reduced photosynthesis, reducing the aesthetic of waterbodies, and bioaccumulating in soils where they are a threat to living organisms, including humans, due to their carcinogenic nature [2,3]. Another severe global threat that is of concern at the moment is the emergence of antibiotic-resistant bacteria, which is a threat to public healthcare. Additionally, there has been a surge in illnesses due to bacterial contamination in the food industry, making it a public health concern [4,5]. Therefore, urgent, affordable, eco-friendly, and sustainable solutions are required to mitigate these serious global threats to public health and the environment.

Nanotechnology offers a potential solution to these critical global issues using nanoparticles (NPs) as photocatalysts in water remediation and antibacterial agents. Among NPs, the properties of ZnO NPs, which include high chemical sensitivity, high thermal stability, high electron mobility, wide bandgap, non-hazardous, antimicrobial, etc., have allowed their use in applications such as photocatalysis, biomedicine [6], solar cells [7], gas sensing [8], etc. Green-synthesized ZnO NPs prepared using biological extracts as reducing and stabilizing agents have gained traction in research over the years. Green synthesis stands out as a superior and innovative alternative to conventional methods for producing metal and metal oxide NPs. This technique is not only environmentally friendly and safe but also cost-effective, and remarkably simple, functioning effectively under ambient conditions. Using bio-reductants extracted from plants, this process significantly reduces the environmental impact of NPs production. These natural agents not only facilitate the reduction process but also stabilize the NPs, effectively playing dual roles as both reducing and capping agents. Moreover, green-synthesized ZnO NPs are non-toxic, biocompatible, and have the potential for large-scale production [9,10].

Literature reports have indicated that extracts from plants such as *Camellia sinensis* [11], *Elaeocarpus Sylvestris* [12], *Girardinia diversifolia* [13], *Phoenix roebelenii* (*P. roebelenii*) [14], etc., under different synthesis conditions have successfully been utilized to produce ZnO NPs with remarkable antibacterial and photodegradation activities. Aldeen et al. [14] synthesized ZnO NPs using *P. roebelenii* extracts for antibacterial and photodegradation applications. The ZnO NPs exhibited remarkable photodegradation efficiency of 98.0% in 105 min and significant antibacterial against *Staphylococcus aureus* (*S. aureus*), *Streptococcus pneumoniae*, *Escherichia coli* (*E. coli*) and *Salmonella typhi*. Additionally, several studies have reported better photocatalytic and antibacterial activities against a wide range of dyes and bacteria compared to conventional-prepared ZnO NPs [15]. For example, Fu and Fu [16] reported significantly higher photodegradation activity of green-synthesized ZnO NPs prepared using *Plectranthus amboinicus* extracts (92.5%) compared to chemically synthesized ZnO NPs (67.5%) against Methyl red dye. Hence research efforts are focused on the utilization of green-synthesized ZnO NPs in photodegradation and antibacterial applications.

Both photodegradation and antibacterial activities of plant-mediated ZnO NPs are affected by properties such as crystallinity, optical bandgap, shape, particle size, etc. These properties can be controlled during the synthesis process of NPs by varying synthesis parameters such as annealing temperature, pH, precursor concentration, etc., [17]. Annealing is a post-synthesis process that induces structural and morphological changes in NPs, and it can be used to control the properties of NPs [18]. However, to the best of our knowledge, a few research studies have focused on the influence of synthesis parameters of green-synthesized ZnO NPs. Kem et al. [19] synthesized ZnO NPs using *Citrus limon* (*C. limon*) juice annealed at different temperatures (600 to 900 °C). The SEM results revealed average particle sizes of 64 and 77 nm for *C. limon*-mediated ZnO NPs annealed at 700 and 900 °C, respectively. Sedefoglu [20] reported different properties and photocatalytic activity of *Myrtus communis*-mediated ZnO NPs annealed at various temperatures. These studies demonstrate that annealing temperature can influence the properties and performance of ZnO NPs, and hence, studying the influence is crucial in the development of plant-mediated ZnO NPs and driving towards large-scale production.

Therefore, this study reports for the first time the influence of annealing temperature on the properties of ZnO NPs using biological extracts from *Leonotis ocymifolia* (L.O.) plant obtained from the Eastern Cape province of South Africa. To the best of our knowledge, this is the first time the effect of annealing temperatures on the L.O-mediated ZnO NPs is being reported. L.O. is a medicinal plant that has not been extensively studied and utilized. It has been reported to possess biomolecules such as saponins, alkaloids, terpenoids, and flavonoids that can be utilized during the green synthesis of ZnO NPs to reduce and stabilize the NPs [21]. Additionally, this work reports for the first time the influence of annealing temperatures on the application of the L.O-mediated ZnO NPs for the photocatalytic and antibacterial activities against methylene blue (MB) dye and *E. coli* and *S. aureus*. The findings of this study show that annealing temperature affects the properties of plant-mediated ZnO NPs and their performance as antibacterial agents and photocatalysts.

## 2. Materials and Methods

### 2.1. Plant Collection and Preparation

L.O. leaves were collected from Cefani Nursery in Cintsa West, East London, South Africa, and were washed several times with tap water, followed by distilled water to remove dust and other foreign objects. The leaves were then air dried for 5 days to remove moisture and ground to powder using an electric blender. The L.O. extracts were prepared by adding 25 g of L.O. powder to 500 mL of distilled water and heating the mixture at 80 °C for 1 h. The mixture was allowed to cool to room temperature (RT) and filtered using Whatman No. 1 filter paper to collect the L.O. extracts. The L.O. extracts were stored in the refrigerator at 4 °C until further use.

### 2.2. Synthesis of ZnO NPs

The synthesis of the L.O-mediated ZnO NPs was carried out by adding 40 mL of L.O. extracts to 0.2 M solution of zinc chloride (reagent grade, 98%, Merck, South Africa), and the pH was adjusted to 12 using sodium hydroxide (reagent grade, 98% pellets, Merck, South Africa). The mixture was then heated at 60 °C for 2 h. The mixture was centrifuged at 6000 rpm for 20 min and washed several times with distilled water and ethanol (reagent grade, ≥99.5, Merck, South Africa) to remove unreacted zinc chloride, sodium hydroxide, and L.O. extracts. The obtained precipitate was dried in an oven at 60 °C overnight and then annealed at 350, 550, and 750 °C to obtain the L.O-mediated ZnO NPs, which were labeled 350, 550, and 750, respectively. The unannealed ZnO NPs were labeled 000.

### 2.3. Characterization

The L.O-mediated ZnO NPs were characterized using a powder X-ray diffraction (XRD) Rigaku Miniflex600 (Cukα), Germany with a scan range of 10–90°, a scan rate of 0.01 min^−1^, and an electron energy of 40 keV. Fourier transform infrared (FTIR) spectra were obtained using a PerkinElmer Spectrum Two, United Kingdom with a wavelength range of 400–4000 cm^−1^ and 1 cm^−1^ resolution. The optical studies were performed using an ultraviolet-visible spectroscopy (UV–Vis) HACH DR6000, South Africa. The surface morphology and elemental analysis were carried out using scanning electron microscopy equipped with an energy dispersive X-ray spectrometer (EDS), SEM-EDS TESCAN VEGA model from JEOL, Peabody, MA, USA, with an accelerating voltage of 2 kV. The surface area and pore sizes were analyzed using nitrogen adsorption-desorption analysis Brunauer-Emmett-Teller (BET) using a Micromeritics Tristar II 3020, USA, at a pretreatment temperature of 150 °C and a run time of 300 min.

### 2.4. Photodegradation Studies

The photodegradation studies were performed using an 18 W UVB light and MB dye as the model dye, following a method reported by Mugumo et al. [22]. An amount of 100 mg L.O-mediated ZnO NPs was added to a 100 mL solution of 10 ppm in a 250 mL beaker and placed on a magnetic stirrer. The mixture was stirred in the dark for 30 min to establish the adsorption-desorption equilibrium. The photodegradation experiment was conducted for 180 min and 2 mL was drawn using a syringe every 30 min. The mixture was centrifuged at 10,000 rpm, and the absorbance was determined using a UV–Vis Biochrom WPA Lightwave II spectrophotometer at λ_max_ = 663 nm. The efficiency was calculated using Equation (1).

(1) $$\%\;degradation=\frac{C_{0}-C}{C_{0}}\times 100$$

where *C*_0_ is the initial dye concentration at time = 0, and *C* is the dye concentration at time = t.

The photodegradation of MB dye using L.O-mediated ZnO NPs was optimized by varying pH, dosage, and initial dye concentration as follows: pH: 3, 5, 9, and 11, photocatalyst dosage: 40, 80, 100, and 120 mg, and initial dye concentration:10, 15, 20, and 25 ppm MB dye.

### 2.5. Antibacterial Studies

The L.O-mediated ZnO NPs were investigated for antibacterial activity against Gram-positive (G+) *S. aureus* and Gram-negative (G−) *E. coli* bacteria. The antibacterial studies were performed using the agar well diffusion method. The method involved first growing the bacteria in nutrient growth media overnight. The bacterial inoculum was then standardized to 0.5 McFarland units. The L.O-mediated ZnO NPs, negative control, and positive control (ciprofloxacin) were added to the 6 mm deep discs of the plates inoculated with bacteria and incubated at 37 °C for 18 h in the dark. The tests were carried out in duplicates and the ZOI, which are measured in mm and indicate the antibacterial potency.

## 3. Results and Discussion

### 3.1. Structural Analysis

**Figure 1:**
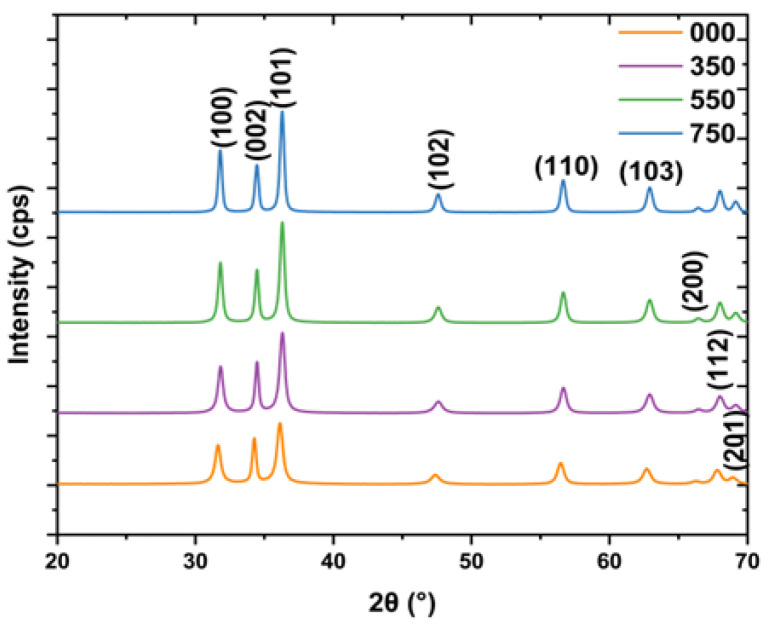
XRD patterns of L.O-mediated ZnO NPs synthesized at different temperatures.

Powder XRD is a non-destructive tool commonly utilized to study the structure of NPs. The XRD diffraction patterns of L.O-mediated ZnO NPs annealed at different temperatures are depicted in Figure 1. The diffraction patterns confirmed the presence of a hexagonal wurtzite crystal structure belonging to the space group P*63mc*. In the hexagonal crystal structure, the Zn^2+^ and O^2−^ are interconnected sublattices, with each Zn^2+^ surrounded by four tetrahedral and O^2−^ and vice-versa. All the diffraction peaks of 000, 350, 550, and 750 can be indexed to (100), (002), (101), (102), (110), (103), (200), (112), and (201) in order of increasing 2θ as supported by the Joint Committee on Powder Diffraction Standards (JCPDS) Card Number: 36–1451. No extra peaks were detected, which confirmed the presence of high-phase purity ZnO NPs. This indicated that the biomolecules from the L.O. extracts effectively reduced the zinc chloride precursor to ZnO NPs and stabilized the synthesized NPs. Thus, the synthesis of ZnO NPs using L.O. extracts has the potential to produce high-quality NPs in an economical and eco-friendly manner that can be used for various applications.

The crystallite sizes (D) were estimated using the Scherrer equation given in Equation (2).

(2) $$D=\frac{0.9\lambda }{\beta cos\theta }$$

where *λ* is the X-ray wavelength (0.154 nm), *θ* is Bragg’s diffraction angle, and *β* is the width at half-maximum intensity (FWHM).

The d spacing and the lattice constants ‘a’ and ‘c’ for the (100) (002) and (101) planes were calculated using Equations (3)–(5) below.

(3) $$d=\frac{\lambda }{2sin\theta }$$

(4) $$a=\frac{\lambda }{\sqrt{3}sin\theta }$$

(5) $$c=\frac{\lambda }{sin\theta }$$

The average crystallite sizes of 000, 350, 550, and 750 were 13.8, 14.9, 17.4, and 20.4 nm, respectively. The crystallite sizes increased with an increase in annealing temperature. This can be attributed to the coalesces of smaller crystallites into larger and more thermodynamically stable crystallites and reduced grain defects [23]. The FWHM is often used as an indicator of crystal regularity or crystallite size [24]. The FWHM decreased with an increase in annealing temperature, as seen in Table 1, and is also supported by the increase in the sharpness of XRD diffraction peaks that can be observed from the XRD patterns in Figure 1. For example, the FWHM values for the (100) plane were 0.611, 0.532, 0.427, and 0.360 for 000, 350, 550, and 750, respectively, which indicated that crystallinity improved with an increase in the annealing temperature. An increase in annealing temperature increases the energy of atoms, and this allows the migration of atoms to the correct lattice positions, leading to better-orientated crystals with reduced defects [25]. Uthirakumar and Hong [26] also reported a decrease in FWHM values and an increase in diffraction peak sharpness with an increase in the annealing temperature (100 to 300 °C) of ZnO NPs, which they attributed to the improved crystallinity of the synthesized ZnO NPs. The annealing temperature significantly influences the properties of L.O-mediated ZnO nanoparticles, particularly their crystallinity and crystallite size. These characteristics are crucial as they directly impact the effectiveness of the NPs applications such as photodegradation.

### 3.2. FTIR Analysis

**Figure 2:**
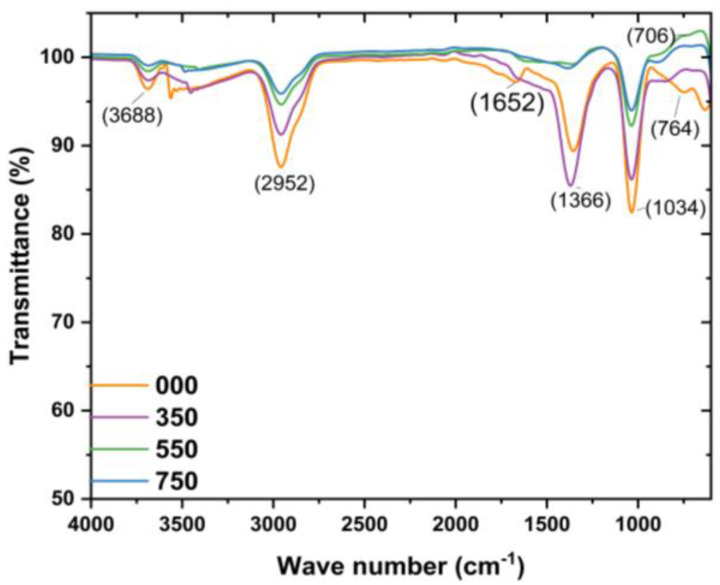
FTIR spectra of L.O-mediated ZnO NPs annealed at different temperatures.

FITR is an important tool that uses vibrational states to recognize the specific bonds of materials and is employed to reveal the functional groups from the reducing and stabilizing L.O. biomolecules on the surface of the L.O-mediated ZnO NPs in the region 400–4000 cm^−1^. The FTIR spectra of the L.O-mediated ZnO NPs are given in Figure 2. From the literature, the Zn–O bond stretch vibrations have been reported in the range of 400–800 cm^−1^ and are used to confirm the presence of ZnO [27]. In this study, the peaks observed around 700–764 cm^−1^ were therefore attributed to the Zn and O interactions, confirming the formation of ZnO NPs using the biomolecules from the L.O. extracts. This is similar to the FTIR observations by Jayarambabu and co-workers [28] for *Curcurm longa* tuber-mediated ZnO NPs. The Zn–O bond vibrations stretch was detected between 731.9 to 608.6 cm^−1^. The FTIR results for the prepared L.O-mediated ZnO NPs are consistent with the XRD findings, which confirmed the synthesis of ZnO NPs using zinc chloride as a precursor and L.O. extracts as the reducing agent as well as the capping agents. The FTIR spectrum of the as-prepared L.O-mediated ZnO NPs revealed peaks at about 1034, 1366, 1652, and 2952 cm^−1^, which can be assigned to C–N stretching of amines, C–H stretch vibrations, C=O stretching vibrations, and C–H stretch vibrations, respectively [28,29]. However, the peak intensity decreased with an increase in annealing temperature with a complete disappearance of peaks at about 1366 and 1652 cm^−1^, which suggests the decomposition of the L.O. molecules present on the surface of the ZnO NPs.

### 3.3. Optical Analysis

**Figure 3:**
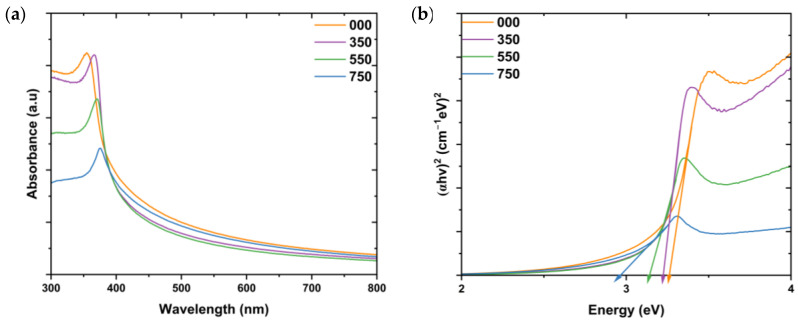
(**a**) UV–Vis spectra and (**b**) the Tauc plots of the L.O-mediated ZnO NPs synthesized at different annealing temperatures.

ZnO NPs can absorb electromagnetic radiation in the UV and near-visible regions, with absorption characteristics influenced by particle size, crystallite size, and shape. This absorption is primarily due to ZnO’s intrinsic bandgap, with reported absorption peaks typically falling between 310 and 380 nm [30]. The characteristic peak of L.O-mediated ZnO NPs annealed at different temperatures was investigated using UV–Vis, and the results are depicted in Figure 3a.

From the graph, it can be seen that all samples exhibited a single strong absorption peak in the region between 300 and 400 nm. The absorption peaks of the synthesized L.O-mediated ZnO NPs were observed at 354, 366, 370, and 375 nm for 000, 350, 550, and 750, respectively. The absorption peaks shifted to longer wavelengths as the annealing temperature increased. This can be attributed to the reduction in crystallite sizes with an increase in annealing temperature, as supported by the XRD results. These results align with findings by Umar et al. [31], who observed a shift of the absorption peaks to higher wavelengths (374 to 382 nm) with an increase in annealing temperatures (400 to 800 °C) of ZnO NPs synthesized by facile combustion method.

The bandgaps of the L.O-mediated ZnO NPs were estimated using the Tauc plot using Equation (6) below.

(6) $$ahv=A{\left(hv-E_{g}\right)}^{2}$$

where *hv* is the energy of the incident photon, *A* is a constant, α is the absorption coefficient, and *E_g_* is the bandgap. The bandgaps were estimated using the x-intercept from extrapolation of the linear portion of the plot of (*αhv*)^2^ vs. hv, as shown in Figure 3b. The bandgaps were 3.27, 3.22, 3.14, and 2.95 eV for 000, 350, 550, and 750, respectively. The smaller bandgap of 750 means that less energy is required for the transition of electrons from one energy state to another, making them ideal for photocatalysis. The bandgaps of the L.O-mediated ZnO NPs annealed at different temperatures decreased with an increase in annealing temperature. This is in contrast with the UV–Vis results reported for ZnO NPs synthesized using chemical sol–gel and annealed at 200, 500, and 700 °C. The calculated bandgaps were 3.33, 3.42, and 3.45 eV, respectively, which was attributed to quantum confinement effects [32]. In this work, the bandgap decreased due to an increase in the crystallite sizes as the annealing temperature increased. Additionally, the decrease in bandgap can be a result of an increase in surface dangling bonds due to crystallite breakdown at high temperatures, which, therefore, increases the localized states. Dangling bonds are produced from the crystallization process during the annealing process [33]. Chan and associates [34] also observed a decrease in bandgap with an increase in the annealing temperature of *Garcinia mangostana*-mediated ZnO NPs. The bandgap dropped from 3.27 to 2.80 eV with an increase in the annealing temperature from 300 to 500 °C.

### 3.4. Morphological and Elemental Analysis

**Figure 4:**
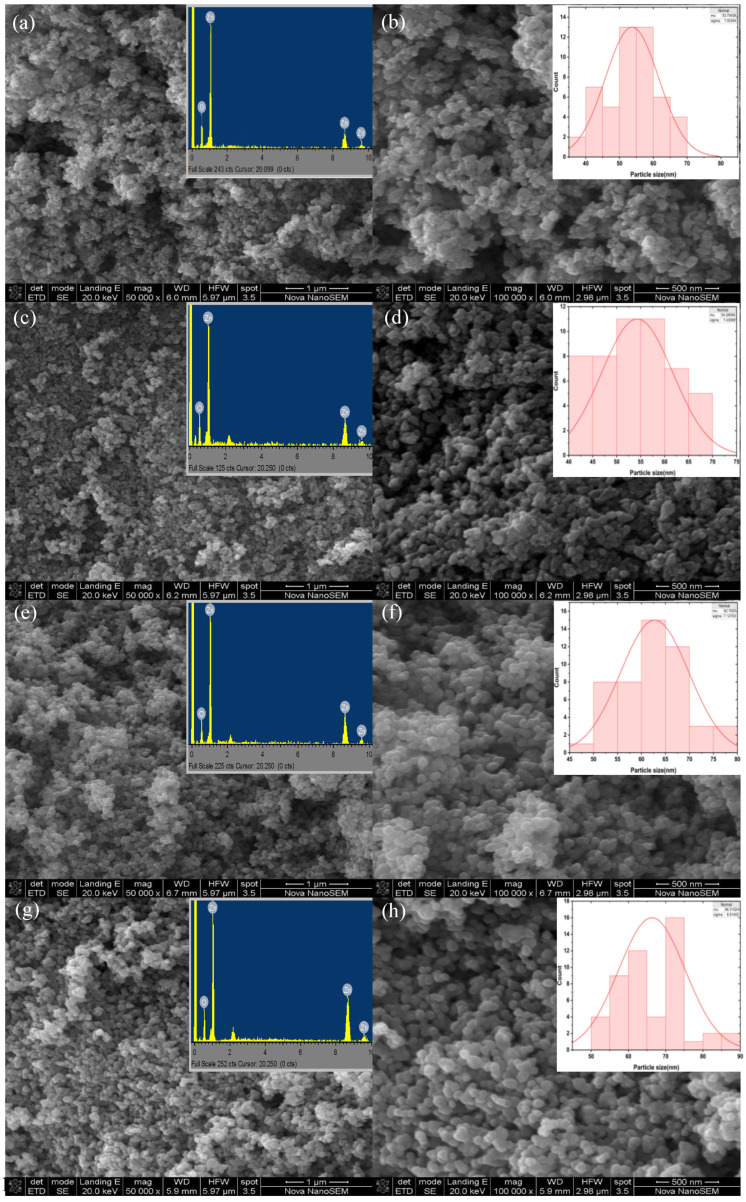
SEM images of L.O-mediated ZnO NPs annealed at different temperatures at low magnification (**a**) 000, (**c**) 350, (**e**) 550, and (**g**) 750. EDS spectra of the synthesized L.O-mediated ZnO NPs annealed at different temperatures Insets (**a**) 000, (**c**) 350, (**e**) 550, and (**g**) 750. SEM images of L.O-mediated ZnO NPs annealed at different temperatures at high magnification (**b**) 000, (**d**) 350, (**f**) 550, and (**h**) 750. Particle size distribution graphs of the synthesized L.O-mediated Insets (**b**) 000, (**d**) 350, (**f**) 550, and (**h**) 750.

Antibacterial and photodegradation activity is dependent on properties of NPs, such as shape and particle size. The morphology of the L.O-mediated ZnO NPs was studied using SEM and the SEM micrographs are given in Figure 4. The SEM micrographs in Figure 4b,d,f,h revealed that the shapes of the L.O-mediated ZnO NPs as irregular and semi-spherical for 000, semi-spherical for 350 and 550, and mostly spherical for 750. All the synthesized ZnO NPs exhibited some agglomeration, a typical feature of plant-mediated ZnO NPs. Additionally, the SEM images revealed that the morphology of the particles became more pronounced and well-defined as the annealing temperature increased. The lack of well-defined spherical particles in 000 could be ascribed to large quantities of biomolecule moieties on the surface, as supported by the higher peak intensity from the FTIR spectra compared to 350, 550, and 750. When the annealing temperature increased, the amount of biomolecule moieties on the surface was reduced, and therefore, their interference on the surface, which results in structural defects, was reduced, allowing for more defined particles [35]. These findings demonstrate the importance of annealing NPs as they influence their properties. The average particle size was estimated using Image Processing and Analysis in Java (Image J, version 1.54k) Java 8 software. The average particle sizes of 000, 350, 550, and 750 were 53.7, 54.3, 62.8, and 66.3 nm, respectively, which were higher than the calculated crystallite sizes from the XRD analysis. However, the particle size increased with an increase in annealing temperature which is in agreement with the XRD crystallite sizes trend.

The elemental composition of the synthesized ZnO NPs was analyzed using EDS, and the micrographs are given in insets in Figure 4a,c,e,g. The EDS micrographs confirmed the formation of ZnO NPs from L.O. extracts as indicated by the three peaks corresponding to Zn about 1.0, 8.7, and 9.5 keV, with the prominent peak at 1.0 keV and a peak of O at about 0.5 keV. This demonstrates that the biomolecules in the L.O. extracts successfully reduced zinc chloride to ZnO NPs. No other peaks were detected in the EDS micrographs, which showed that the synthesized ZnO NPs had no impurities. These results were in conformity with the obtained XRD results which revealed the presence of high-phase purity ZnO NPs. Additionally, according to the EDS analysis, the weight percentage ratios of Zn:O were 79.1:20.7, 77.5:22.5, 78.1:21.9, and 79.7:20.1 for 000, 350, 550, and 750, respectively. These weight percentages were in close agreement with the ratio of bulk ZnO (80:20) [36] and this further confirms the formation and purity of the synthesized ZnO NPs using L.O. extracts.

### 3.5. Brunauer, Emmett and Teller (BET) Analysis

**Figure 5:**
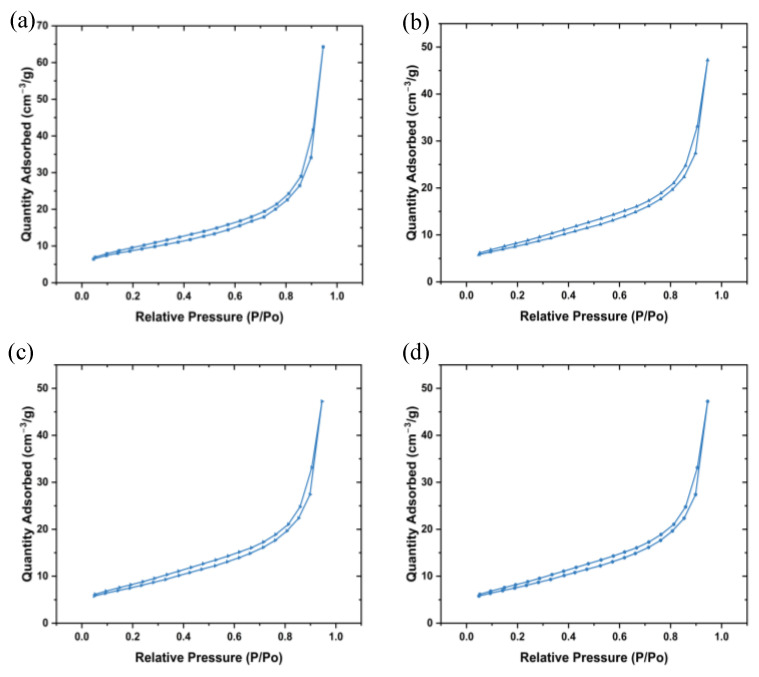
N_2_ adsorption-desorption isotherms of the synthesized ZnO NPs (**a**) 000, (**b**) 350, (**c**) 550, and (**d**) 750.

BET is an approach for determining the surface area and pore size distribution of materials based on gas adsorption that was developed by Brunauer-Emmett-Teller. BET takes into account the multilayer adsorption of non-corrosive gases such as N_2_ and Ar on the surface of the NPs [37]. This is important in photocatalysis and antibacterial applications due to these reactions occurring on the surface of the NPs. Figure 5a–d show the plotted N_2_ adsorption-desorption isotherms of the synthesized ZnO NPs. All the plots followed the type III isotherm according to the International Union of Pure and Applied Chemistry (IUPAC) [38]. The BET surface area of 000, 350, 550, and 750 were 31.6, 27.5, 16.9, and 13.2 m^2^/g, respectively, and decreased with an increase in annealing temperature as seen in Table 2. These findings agree with the obtained XRD and SEM results, which revealed an increase in crystallite and particle size along with an increase in annealing temperature. Additionally, the pore volume decreased with an increase in the annealing temperature which could be owing to the collapsing of pores due to agglomeration of particles as the annealing temperature increases [39].

### 3.6. Photocatalytic Activity of L.O-Mediated ZnO NPs

**Figure 6:**
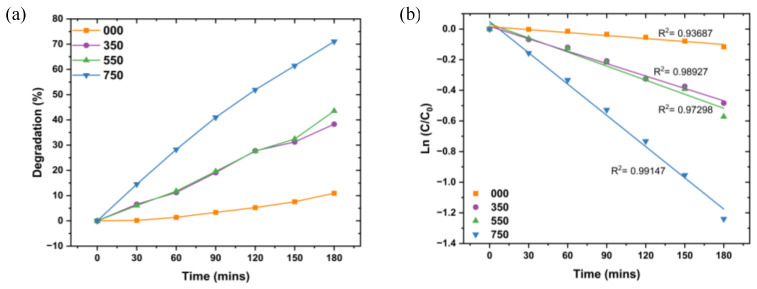
(**a**) % Removal of L.O-mediated ZnO NPs annealed at different temperatures. (**b**) Kinetics study of the degradation of MB dye using L.O-mediated annealed at different temperatures.

Photodegradation is an environmentally friendly wastewater treatment technology that utilizes light radiation to degrade pollutants such as dyes. The treatment technology is based on the absorption of energy that is greater or equal to the bandgap of photocatalysts when exposed to light irradiation. The absorption of energy results in the excitation of electrons from the valence band (VB) to the conduction band (CB), resulting in the creation of charge carriers (electron–hole pairs). These charge carriers take part in redox reactions on the surface of the photocatalyst, generating reactive oxygen species (ROS) such as hydroxyl radical and superoxide radical, which are responsible for the degradation of dyes [40]. In our work, L.O-mediated ZnO NPs were employed as photocatalysts to degrade MB dye under UV radiation and in 180 min. The degradation efficiencies of the photodegradation of MB dye using 000, 350, 550, and 750 with an increase in time are given in Figure 6a.

The degradation efficiencies were 11.0, 38.2, 43.6, and 71.1% for 000, 350, 550, and 750, respectively. The degradation efficiencies increased with an increase in annealing temperature. The more significant photocatalytic activity of 750 despite larger crystallite size, larger particle size, and lower BET surface area than 000, 350, 550, and 750 can be attributed to the smaller bandgap of 2.95 eV compared to 000, 350, and 550. The smaller bandgap allows the absorption of photons with less energy to generate charge carriers and promotes the generation of charge carriers, which are critical in photodegradation [41]. The well-defined spherical shape of 750 compared to 000, 350, and 550 could have resulted in a difference in photocatalytic performances as it improves the mobility of electron–hole pairs and charge separation, which reduces electron–hole recombination [42] Additionally, it had higher crystallinity compared to 000, 350, and 550, which results in reduced defects. Defects can act as recombination sites and this reduces the charge carriers available to generate ROS, consequently leading to reduced photocatalytic activity [43]. Narath and associates [44] also reported an increase in degradation efficiency with an increase in annealing temperature for the photodegradation of MB dye using *Cinnamomum tamala*-mediated ZnO NPs. The degradation efficiencies were reported to be 82.4, 87.5, and 93.9% for the ZnO NPs annealed at 300, 500, and 700 °C, respectively, in 90 min.

The kinetics studies of L.O-mediated ZnO NPs for the degradation of MB dye were estimated using the pseudo-first-order kinetics equation shown in Equation (7).

(7) $$In\frac{C}{C_{0}}=-kt$$

where *C* is the concentration of MB dye at time (*t*), *C*_0_ is the concentration of MB dye at *t* = 0, *t* is the time, and *k* is the rate constant at time (*t*).

The rate constants were estimated from the linear plot of ($In\frac{C}{C_{0}}$) vs. time (*t*) and *k* given by the slope of the linear plot. As seen in Figure 6b, the photodegradation of MB dye using the L.O-mediated ZnO NPs is in agreement with the pseudo-first-order model, with R^2^ values > 0.90. The rate constants increased with an increase in annealing temperature, with rate constants of 6.4 × 10^−4^, 2.7 × 10^−3^, 3.1 × 10^−3^, and 6.8 × 10^−3^ min^−1^ for 000, 350, 550, and 750, respectively. A temperature of 750 °C had the highest rate constant owing to its structural, morphological, and optical properties and, therefore, was used in the optimization studies of the degradation MB dye using L.O-mediated ZnO NPs.

#### 3.6.1. Optimization of the Photodegradation of MB Dye Using L.O-Mediated ZnO NPs

**Figure 7:**
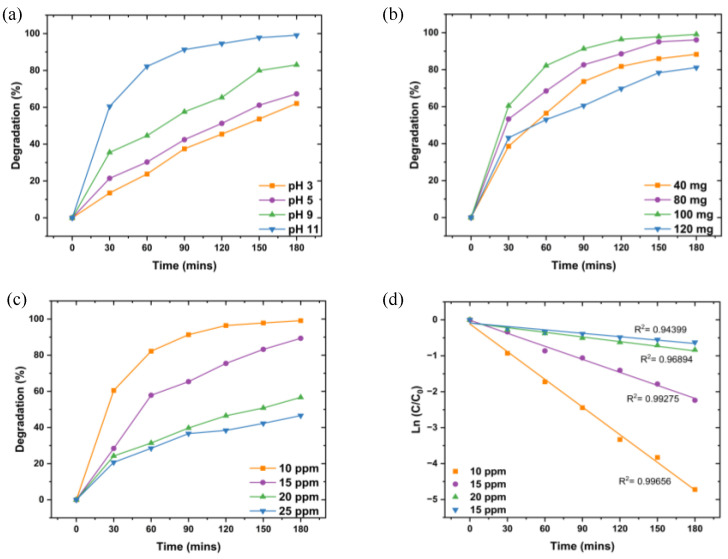
The graphs of degradation efficiencies of MB dye using L.O-mediated at different (**a**) pHs, (**b**) dosages, (**c**) initial MB dye concentration, and (**d**) Kinetics study of the degradation of MB dye using L.O-mediated ZnO NPs.

Photodegradation can be influenced by reaction parameters such as pH, initial dye concentration, and photocatalyst dosage; therefore, optimizing these parameters is crucial to ensure maximum degradation. The effect of pH was studied at 100 mg L.O-mediated ZnO NPs dosage, 10 ppm initial MB dye concentration, varying pH from 3 to 11, and under UV irradiation for 180 min. The degradation efficiencies of L.O-mediated ZnO NPs against MB dye at varying pHs are illustrated in Figure 7a. The degradation efficiency increased with an increase in pH, with the lowest degradation at pH 3 (62.1%) and the highest degradation at pH 11 (99.1%). As a result, pH 11 was used as the optimal pH for the degradation of MB dye using the prepared L.O-mediated ZnO NPs. The point of zero charge of ZnO is at pH 9 [45]. Hence, at pH higher than 9, the surface of ZnO is negatively charged and interacts with the positively charged molecules of MB dye owing to electrostatic interactions, which enhances the degradation efficiency. The surface of the L.O-mediated is positively charged at low pH, resulting in fewer hydroxyl ions for the generation of hydroxyl radicals, which inherently reduces the photodegradation efficiency.

The effect of dosage was studied using 100 mL of 10 ppm MB dye initial concentration, pH 11, varying dosage from 40 to 120 mg, and UV irradiation for 180 min. The degradation efficiencies of the influence of L.O-mediated ZnO NP dosage are shown in Figure 7b. Increasing the L.O-mediated ZnO NP dosage from 40 to 100 mg led to an increase in degradation efficiency (88.3 to 91.1%, respectively); however, a further increase to 120 mg resulted in a decrease in efficiency (81.1%). The increase in degradation efficiency with an increase in dosage can be attributed to the increase in available active site sites for the creation of electron–hole pairs and, consequently, higher generation of hydroxyl radicals, which subsequently leads to improved photocatalytic activity. However, a further increase in dosage results in a decrease in degradation efficiency owing to increased turbidity which reduces light penetration and light scattering, therefore leading to reduced charge carrier generation and hence reduced efficiency [46].

The effect of initial MB dye concentration on degradation efficiency was evaluated at pH 11 using 100 mg ZnO NPs, with concentrations varying from 10 to 25 ppm. As shown in Figure 7c, degradation efficiency decreased with increasing dye concentration, from 91.1% at 10 ppm to 46.6% at 25 ppm. Despite this decrease in percentage efficiency, the absolute amount of dye removed was greater at higher concentrations, with 11.7 ppm removed from the 25 ppm solution compared to 9.11 ppm from the 10 ppm solution. This trend may result from the limited number of active sites on the ZnO NPs, which can become saturated at higher concentrations, along with reduced light penetration due to the higher concentration of dye molecules. Therefore, 10 ppm was chosen as the optimal concentration for maximizing degradation efficiency.

Kinetics studies of initial MB dye concentrations (Figure 7d) showed that degradation followed the Langmuir-Hinshelwood pseudo-first-order model (R^2^ > 0.90), with rate constants decreasing as concentration increased (2.6 × 10^−2^, 1.2 × 10^−2^, 4.3 × 10^−3^, and 3.2 × 10^−2^ min^−1^ for 10, 15, 20, and 25 ppm, respectively). The decreased rate constants at higher concentrations suggest a reduction in active surface sites and limited light penetration, which hinders dye molecule interactions with the photocatalyst.

#### 3.6.2. Recyclability Studies

**Figure 8:**
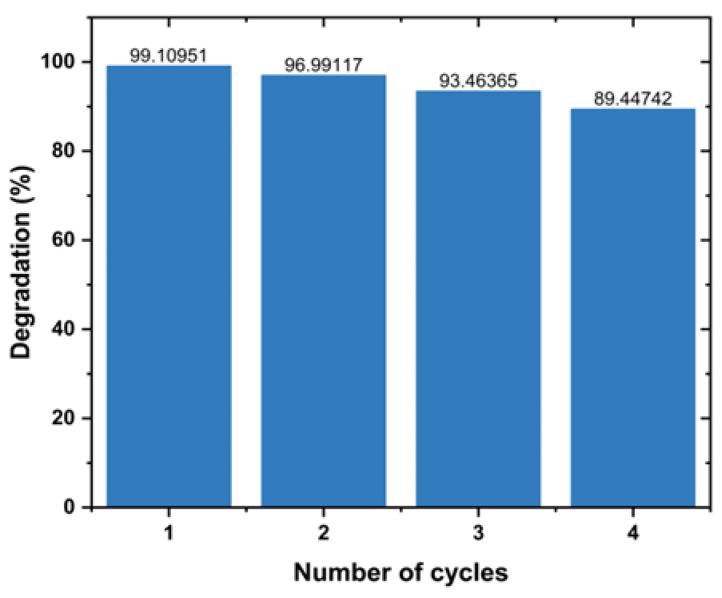
Degradation efficiency of MB dye using L.O-mediated after 4 cycles.

The recyclability of L.O-mediated photocatalyst for the degradation of MB dye was assessed over four degradation cycles. The experiments involved centrifuging, washing with distilled water, and drying the NPs in the oven after each cycle. Each cycle was carried out at pH 11, dosage of 100 mg, 10 ppm initial MB dye concentration and under UV exposure for 180 min. Figure 8 depicts the degradation efficiency of MB dye using L.O-mediated ZnO NPs per cycle. The prepared L.O-mediated ZnO NPs exhibited high stability, as demonstrated by a reduction in efficiency of about 10% after four cycles. The reduction in efficiency can be attributed to the loss of active sites due to the adsorption of degradation products. This limits the active sites available for the generation of electron–hole pairs and, hence, the production of hydroxyl radicals, resulting in a reduction in efficiency. These findings suggest that L.O-mediated ZnO NPs are stable photocatalysts that can be used to effectively degrade dyes into less toxic products. Aziz et al. [47] also reported highly stable ZnO NPs prepared using *Grewia asiatica* extract. The degradation efficiency of MB dye using the synthesized ZnO NPs decreased from 92 to 89% after four cycles under solar radiation for 70 min.

### 3.7. Antibacterial Activity of L.O-Mediated ZnO NPs

The antibacterial activity of L.O-mediated ZnO NPs annealed at different temperatures was evaluated against G+ *S. aureus* and G− *E. coli* bacteria using the agar well diffusion method. Antibacterial efficacy was assessed by measuring the diameter of the zone of inhibition (ZOI) in millimeters (mm), indicating the potency of bacterial growth inhibition. ZnO NPs are known to exhibit antimicrobial activity through electrostatic interactions between the positively charged NPs and negatively charged bacterial membranes, leading to cell membrane disruption and leakage of cytosolic contents. Additionally, Zn^2+^ ions from ZnO NPs can penetrate bacterial cells, bind to amino acids, disrupt DNA replication, and interfere with the electron transport chain, ultimately causing cell death [48].

The antibacterial efficacy of the prepared L.O-mediated ZnO NPs decreased with increased annealing temperature, as demonstrated by a reduction in ZOI against *S. aureus* from 15 mm (unannealed) to 7 mm (750 °C) at a 5 mg/mL ZnO NPs dosage. The ZOI of the synthesized ZnO NPs, positive control, and negative control against *S. aureus* and *E. coli* are given in Table 3.

This higher antibacterial activity in the unannealed sample can be attributed to its smaller particle size and larger surface area, as observed in SEM and BET analyses, which enhance interaction with bacterial cells. These findings suggest that smaller particle sizes and higher surface area facilitate stronger interactions with bacteria, therefore increasing antibacterial potency.

**Figure 9:**
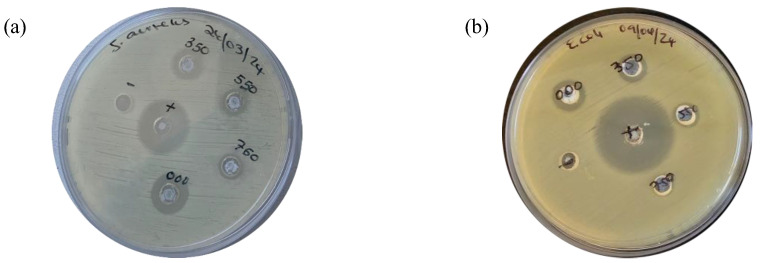
ZOI of the synthesized ZnO NPs, positive control, and negative control against (**a**) *S. aureus* and (**b**) *E. coli*.

In comparison, the prepared ZnO NPs exhibited reduced antibacterial activity against *E. coli* compared to *S. aureus* as seen in Figure 9. The unannealed L.O-mediated ZnO NPs displayed a ZOI of 12 mm against *E. coli*, while the sample annealed at 750 °C showed negligible activity.

This difference in susceptibility between G+ and G− bacteria is likely due to the presence of hydrophobic lipopolysaccharides in the outer membrane of G− bacteria, which impedes NP penetration and reduces antibacterial efficacy [49]. Similar findings by Aziz et al. [50] also reported a decrease in antibacterial activity with an increase in annealing temperature for *Anchusa italica*-mediated ZnO NPs annealed at 100 and 200 °C. The authors also observed greater antibacterial against G+ bacteria than G− bacteria. The reduced antibacterial activity of the synthesized ZnO NPs against *E. coli* can be attributed to the presence of hydrophobic lipopolysaccharide in the outer membrane that envelopes the G− bacteria cells, which makes it difficult for NPs to penetrate. This results in reduced sensitivity of G− bacteria to antibacterial agents.

## 4. Conclusions

ZnO NPs were synthesized successfully using a relatively cheap and environmentally friendly route using L.O. leaf extracts as the reducing and capping agent. The effects of annealing temperature on the structural, optical, and morphological properties of the synthesized NPs were studied. Additionally, the effects of these properties of the prepared ZnO NPs in photodegradation and antibacterial applications were also reported. The XRD confirmed the synthesis of ZnO NPs with a hexagonal wurtzite structure. In addition, XRD revealed that crystallinity and crystallite sizes increased with an increase in annealing temperature, with the calculated average crystallite sizes of 13.8 to 20.4 nm. The calculated bandgap from UV–Vis results were observed to decrease (3.27 to 2.95 eV) with an increase in annealing temperature due to an increase in crystallite size. SEM results revealed that the L.O-mediated ZnO NPs had well-formed spherical particles than 000, 350, and 550. The average particle size also increased with an increase in annealing, and this was supported by the BET revealed surface area of 31.6, 27.5, 16.9, and 13.2 m^2^/g for 000, 350, 550, and 750, respectively. These findings suggest that annealing temperature impacted the properties of the prepared L.O-mediated ZnO NPs. Annealing temperature of 750 °C exhibited the highest photodegradation efficiency against MB dye under UV exposure for 180 min (71.1%). This was due to the high crystallinity, well-formed morphology, and smaller bandgap compared to 000, 350, and 550. The optimum operational conditions from the optimization studies of the degradation of MB dye using L.O-mediated annealed at 750 °C were pH 11, 100 mg dosage, and 10 ppm initial dye concentrations, with a degradation efficiency of 99.1% in 180 min. The prepared ZnO NPs exhibited high stability after four degradation cycles. The antibacterial studies using the synthesized L.O-mediated ZnO NPs revealed that 000 exhibited better antibacterial activity due to the smaller particle size and higher surface area. According to the degradation studies conducted under UV irradiation and antibacterial tests, annealing temperature significantly impacted the performance of L.O-mediated ZnO NPs. This is demonstrated by the varying degradation efficiencies and antibacterial efficacy at different annealing temperatures, making it a compelling option for various applications such as biomedical and environment remediation. Furthermore, the preparation of ZnO NPs using L.O. extracts is a viable green synthesis route that is affordable and eco-friendly and has potential in other applications.

## Figures and Tables

**Table 1:** Lattice parameters characteristics at stronger diffraction peaks for the (100), (002), and (101) planes of the L.O-ZnO NPs annealed at different temperatures.

Sample	Peak	2θ (°)	FWHM	d (Å)	a (Å)	C (Å)
000	100	31.6	0.6109	2.83	3.26	5.65
350	100	31.8	0.5315	2.81	3.24	5.62
550	100	31.8	0.4272	2.81	3.25	5.62
750	100	31.8	0.3598	2.81	3.25	5.62
000	002	34.3	0.401	2.61	3.02	5.23
350	002	34.5	0.374	2.60	3.00	5.20
550	002	34.5	0.375	2.60	3.03	5.20
750	002	34.5	0.359	2.60	3.00	5.20
000	101	36.2	0.622	2.48	2.87	4.97
350	101	36.3	0.538	2.47	2.86	4.95
550	101	36.3	0.449	2.47	2.86	4.95
750	101	36.3	0.392	2.47	2.86	4.95

**Table 2:** BET results of the synthesized L.O-mediated annealed at different temperatures.

Sample	BET Surface Area (m^2^/g)	Pore Volume (cc/g)	Pore Diameter (nm)
000	31.6	0.0592	12.6
350	27.5	0.0476	10.7
550	16.9	0.0214	7.31
750	13.2	0.0166	6.96

**Table 3:** Antibacterial activity of the synthesized ZnO NPs, positive control, and negative control against *S. aureus* and *E. coli*.

Sample	*S. aureus* (mm)	*E. coli* (mm)
000	15	12
350	11	9
550	9	7
750	7	0
Positive control	20	29
Negative control	0	0

## Data Availability

Data are contained within the article.
